# The Bipolar II Depression Questionnaire: A Self-Report Tool for Detecting Bipolar II Depression

**DOI:** 10.1371/journal.pone.0149752

**Published:** 2016-03-10

**Authors:** Chi Ming Leung, Chi Lap Yim, Connie T. Y. Yan, Cheuk Chi Chan, Yu-Tao Xiang, Arthur D. P. Mak, Marcella Lei-Yee Fok, Gabor S. Ungvari

**Affiliations:** 1 Department of Psychiatry, Shatin Hospital, Hong Kong SAR, China; 2 Unit of Psychiatry, Faculty of Health Sciences, University of Macau, Macao SAR, China; 3 Department of Psychiatry, The Chinese University of Hong Kong, Hong Kong SAR, China; 4 King's College London, King's Health Partners, Department of Psychological Medicine, Institute of Psychiatry, Psychology and Neuroscience, London, United Kingdom; 5 School of Psychiatry and Clinical Neurosciences, University of Western Australia, Perth, Australia; 6 The University of Notre Dame Australia/Marian Centre, Perth, Australia; Catholic University of Sacred Heart of Rome, ITALY

## Abstract

Bipolar II (BP-II) depression is often misdiagnosed as unipolar (UP) depression, resulting in suboptimal treatment. Tools for differentiating between these two types of depression are lacking. This study aimed to develop a simple, self-report screening instrument to help distinguish BP-II depression from UP depressive disorder. A prototype BP-II depression questionnaire (BPIIDQ-P) was constructed following a literature review, panel discussions and a field trial. Consecutively assessed patients with a diagnosis of depressive disorder or BP with depressive episodes completed the BPIIDQ-P at a psychiatric outpatient clinic in Hong Kong between October and December 2013. Data were analyzed using discriminant analysis and logistic regression. Of the 298 subjects recruited, 65 (21.8%) were males and 233 (78.2%) females. There were 112 (37.6%) subjects with BP depression [BP-I = 42 (14.1%), BP-II = 70 (23.5%)] and 182 (62.4%) with UP depression. Based on family history, age at onset, postpartum depression, episodic course, attacks of anxiety, hypersomnia, social phobia and agoraphobia, the 8-item BPIIDQ-8 was constructed. The BPIIDQ-8 differentiated subjects with BP-II from those with UP depression with a sensitivity/specificity of 0.75/0.63 for the whole sample and 0.77/0.72 for a female subgroup with a history of childbirth. The BPIIDQ-8 can differentiate BP-II from UP depression at the secondary care level with satisfactory to good reliability and validity. It has good potential as a screening tool for BP-II depression in primary care settings. Recall bias, the relatively small sample size, and the high proportion of females in the BP-II sample limit the generalization of the results.

## Introduction

Bipolar disorder (BP) is a common psychiatric illness with a lifetime prevalence of approximately 5% in the general population [1, 2] and a point prevalence of 8–10% in primary care settings [3, 4]. In addition to immense suffering for patients and their relatives, BP incurs huge social and economic costs [5–7]. There is increasing evidence that BP is a very disabling illness [8]. A systematic review of 34 papers found that the risk of suicide among BP patients is up to 20–30 times higher than that for the general population [9].

BP depression (BPD), in particular bipolar II disorder (BP-II), is frequently under- or misdiagnosed, mainly for unipolar depression (UPD) [9–12], resulting in suboptimal treatment and poor outcomes [13–16]. Hypomanic symptoms are often neglected or mistaken as highly efficient behaviour or a manifestation of a particular personal style [17, 18].

Techniques to tease out BPD from UPD using fMRI, genetic testing and family studies are being actively explored but remain insufficient [19–21], leaving clinical approaches as the main type of investigation. Several questionnaires or diagnostic schedules targeting the detection of BP have been developed. The Mood Disorder Questionnaire (MDQ) [9], Hypomania Checklist-32 [22] and Bipolar Disorder Screening Scale [23] all include only hypomanic cues for the diagnosis of BP, but do not cater for BPD. The Bipolar Spectrum Diagnostic Scale (BSDS) [24] does include depressive items but has poor positive predictive value [25]. The Bipolar Depression Rating Scale (BDRS) [26] is the only scale that measures BPD, but it is meant for rating symptom severity in patients already diagnosed with BP rather than for detecting BPD.

As there are no clinical pathognomonic features for BPD, a probabilistic approach for diagnosing BP-I depression has been proposed, with operationalized criteria based on a literature review and ‘clinical reasonability’ [27, 28]. However, no corresponding instrument exists for the diagnosis or screening of BP-II depression, and thus the development of such an instrument is imperative.

This study aimed to develop a brief self-report screening tool for BPD, with discriminant validity against UPD and predictive validity for evolution into full-blown bipolar disorder. As BP-I and BP-II depression present differently [29–31], and BP-II represents the more cryptic but relatively well-defined component of the BP spectrum [1, 2], it was decided that BP-II depression should constitute the main theme of the investigation. The ultimate goal was to develop a screening tool that is brief and simple enough that it can be used in psychiatric outpatient clinics, and even in primary care settings where potential patients abound and expertise is lacking.

## Methods

### Development of the self-report screening tool–Bipolar II Depression Questionnaire (BPIIDQ)

#### Literature search

The phenomenology and historical development of the concepts of UPD and BPD [32, 33] were reviewed. Features related to their differences and also those between BP-I and BP-II [34–36] were scrutinized.

#### Inclusion of items

Atypical features associated with BPD such as hypersomnia and appetite increase (DSM IV-TR 2000) [37], suicide attempts [38], family history of BP or completed suicide [39], young age at onset [40], recurrent course [41], post-partum presentation [34], mixed state [17], phobic and obsessive symptoms [39] and substance abuse [42] were considered.

#### Exclusion of items

Hypomanic cues and those more related to BP-I than BP-II, such as a history of hospital admission and psychosis [42, 43], were filtered out. Three other potential items were excluded: history of suicide attempts was excluded because the validity of self-reports can be limited [44], substance abuse because of its low incidence in the Hong Kong Chinese population [45] and psychomotor retardation because it cannot be readily observed during a relatively brief outpatient visit.

#### Devising the text

Features considered specific to BP-II depression were phrased in modern Chinese, with simplicity and brevity emphasized. The text was further checked linguistically by two bilingual experts in Cantonese and Mandarin Chinese (CY and YTX). The first version was named the Bipolar II Depression Questionnaire-Prototype (BPIIDQ-P).

#### Pilot study and text revision

A pilot study was conducted with 10 BPD and 10 UPD subjects who completed the BPIIDQ-P. All subjects had a history of depressive episodes and were in full remission. Following the pilot study, the questionnaire was further edited to reduce ambiguity.

#### Field work

The main part the study was conducted between October and December 2013 in the psychiatric service of Shatin Hospital, which serves a population of approximately 500,000. Within the study period, all ethnic Chinese outpatients attending the clinical team of the principal investigator (CML) aged 18–65 years and diagnosed with a DSM-IV depressive disorder (including BP with a history of depressive episode, major depressive disorder (MDD) and dysthymia (DYS), were consecutively recruited. In each individual case, the clinical diagnosis was made independently by two psychiatrists (CML and CLY). The principal investigator personally followed all of the cases over many years and made diagnoses based on thorough clinical assessments. The other psychiatrist (CLY) made the diagnoses by reviewing the case records. Joint interviews were conducted to resolve diagnostic disagreements. In unresolved cases, a third assessor (CCC) independently administered the Structured Clinical Interview for DSM-IV Disorders (SCID-DSM-IV) [46, 47] to reach a final diagnosis. All three assessors were blind to the results of the BPIIDQ-P. Patients with significant cognitive impairment, e.g. severe learning disability, dementia or cognitive deficits associated with brain damage, and unstable mental state, e.g. severe depression or psychosis, that jeopardized accurate self-report were excluded.

The subjects completed the BPDIIQ-P and the Hospital Anxiety and Depression Scale (HADS) [48, 49] to measure the severity of depressive symptoms. Forty randomly selected subjects repeated the BPDIIQ-P two weeks later, to measure test-retest reliability. Relevant demographic and clinical data were retrieved from case records and the hospital’s computer system. History of suicide attempts, defined by O’Carroll [50], was further confirmed with the respective subjects at follow-up sessions.

The study protocol was approved by the Joint Chinese University of Hong Kong–New Territories East Cluster Clinical Research Ethics Committee. Written informed consent was obtained from each participant. All information was kept in a computer protected by a password. Only the PI (CML) and co-Investigators have access to the data.

### Statistical analysis

Data were analyzed using the Statistical Package for Social Sciences (SPSS) Version 19. The MDD group was divided into recurrent depressive disorder (RDD) and major depressive episode, which was further subdivided into with psychosis i.e. psychotic depression (PD), and without (MDE). Priority in categorization was given to recurrence over presence of psychosis, as in DSM-IV. Categorical variables were analyzed using chi-square, non-parametric continuous data with Mann Whitney U and continuous variables with Student’s t-test. The significance level was set at 0.05. Discriminant analysis was used to extract the best combination of items for differentiating between BP-II and UP. One-step forward selection binary logistic regression was performed to check for the odds ratios and independence of significant items. Items were weighted according to the odds ratios. Different scoring methods were explored with ROC curves to obtain the best cut-off points with optimal sensitivity and specificity. Exploratory factor analysis was performed with the final version of the BPIIDQ-P.

## Results

### Bipolar depression questions

The BPIIDQ-P consists of 13 bipolar depression questions (bdq) grouped into 2 sections (S1 Table). Section A comprises three questions about the patient’s personal and family history, bdq1 (positive family history), bdq2 (onset before 25) and bdq3 (postpartum depression). Section B comprises 10 questions about the course of the illness and symptoms, bdq4 (episodic course), bdq5 (endogeneity), bdq6 (fatigue), bdq7 (panic attacks), bdq8 (social phobia), bdq9 (emotional numbness), bdq10 (hypersomnia), bdq11 (sense of uselessness), bdq12 (agoraphobia) and bdq13 (obsessive ruminations).

### Demographic and clinical data

A total of 320 BP and UP subjects attended the clinic during the study period. Sixteen subjects were excluded for unstable mental state and 6 declined to participate. Thus, 298 subjects entered the study, giving a response rate of 93%. There was no significant difference in age and sex between the excluded and included patients. Initial diagnostic disagreement between the two psychiatrists occurred in 27 (9.1%) subjects. Following a joint interview, consensus was reached in 22 cases, leaving 5 for final decision by SCID-assisted interview. The demographic and clinical data are presented in Table 1.

**Table 1:** Demographic and clinical data of the study sample.

	BP-IID	BP-ID	UPD	BP-IID vs UPD	BP-IID vs BP-ID
Subjects	70	42	186		
Psychiatric diagnosis					
RDD			63 (33.9%)		
MDE			62 (33.3%)		
PD			17 (9.1%)		
DYS			44 (23.7%)		
M/F	15/55	19/23	31/155	NS	P < .01
Age	47.0 ± 11.2	44.1 ± 9.5	46.8 ± 10.5	NS	NS
Education (yr)	11.3 ± 3.0	12.2 ± 3.4	10.2 ± 3.2	NS	NS
Duration of illness (yr)	13.6 ± 7.7	17.6 ± 9.2	9.2 ± 7.2	NS	NS
Suicide attempt	27.7%	25.6%	26.8%	NS	NS
HADS score	8.3 ± 5.5	5.7 ± 4.7	10.0 ± 4.6	p = .05	NS

BP-ID = Bipolar I depression

BP-IID = Bipolar II depression

UPD = Unipolar depression

RDD = Recurrent depressive disorder

MDE = Major depressive episode

PD = Psychotic depression

DYS = Dysthymia

### Discriminant analysis

The results of the discriminant analysis are shown in Table 2. To maximize the number of valid cases, bdq3 –referring to postpartum depression in women–was omitted from the analysis. Bdq1 (positive family history), bdq2 (onset < 25), bdq4 (episodic course) and bdq10 (hypersomnia) were significant independent predictors (p < .05). Bdq7 (panic attacks), bdq8 (social phobia), bdq12 (agoraphobia) and bdq13 (obsessive ruminations) were positively correlated with the discriminant function, but failed to reach statistical significance. These four items helped to maximize the discrimination of BP-IID from UPD. Bdq5 (endogeneity), bdq6 (fatigue), bdq9 (emotional numbness) and bdq11 (sense of uselessness) were weakly or negatively correlated with the discriminant function of the scale.

**Table 2:** Discriminant analysis for differentiating BP-IID from UPD.

	Standardized function coefficients	Correlations between variables and discriminant function
bdq1 (positive family history)	.567*	.621
bdq2 (onset <25)	.324*	.409
bdq4 (episodic course)	.514*	.396
bdq5 (endogenicity)		
bdq6 (fatigue)		
bdq7 (panic attacks)	.143	.181
bdq8 (social phobia)	.008	.199
bdq9 (emotional numbness)		
bdq10 (hypersomnia)	.408*	.358
bdq11 (sense of uselessness)		
bdq12 (agoraphobia)	.173	.264
bdq13 (obsessive rumination)	.175	.132

Negative values were omitted

*significant predictor

BP-IID = Bipolar II depression

UPD = Unipolar depression

bdq = Bipolar depression question

### Logistic regression

The results of the chi-square tests used to test the bdq items that differentiated BP-IID from other depressive disorders and BP-I (p < .05) are shown in Table 3. Bdq items 1, 2, 4 and 10 individually discriminated BP-IID from UPD, with odds ratios of 3.47, 2.00, 2.09 and 2.36, respectively. In the logistic regression, only one item, bdq1 (positive family history), differentiated between the two groups of disorders (p < .000, Exp (B) 5.93, 95% Cl 2.44–14.37).

**Table 3:** Items of the BPIIDQ-8 differentiating BP-II from UPD and its subgroups.

		*bdq item*
BP-II vs	UP	1, 2, 4, 10
	RDD	8, 10#
	MDE	1, 2, 4, 10, 12#
	PD	1, 2, 4
	DYS	1, 2, 7#, 10

chi square, p < .05; #p = .06

BP-IID = Bipolar II depression

UPD = Unipolar depression

RDD = Recurrent depressive disorder

MDE = Major depressive episode

PD = Psychotic depression

DYS = Dysthymia

bdq = bipolar depression question

bdq1 = “positive family history” bdq7 = “panic attacks”

bdq2 = “onset <25” bdq8 = “social phobia”

bdq3 = “postpartum depression” bdq10 = “hypersomnia”

bdq4 = “episodic course” bdq12 = “agoraphobia”

### The 8-item Bipolar II Depression Questionnaire

#### Components and scoring methods, sensitivity and specificity

**Fig 1:**
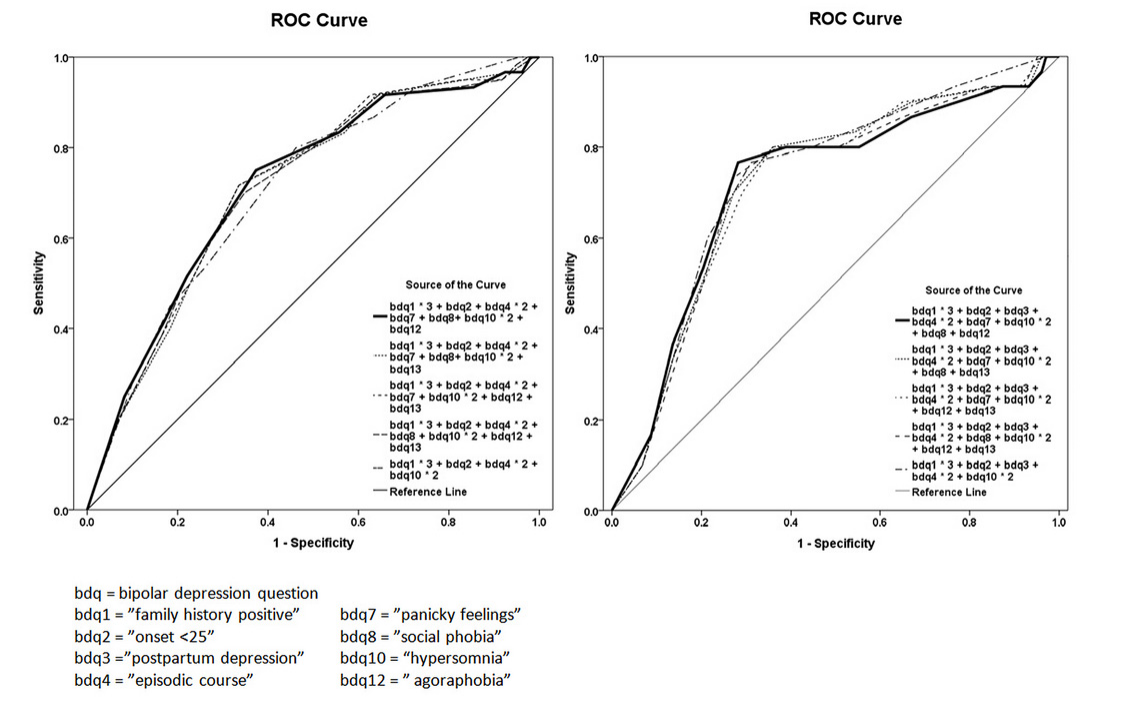
1a. ROC curves for different combinations of bdq items (all cases). Fig 1b. ROC curves for different combinations of bdq items (females with history of childbirth).

**Fig 2:**
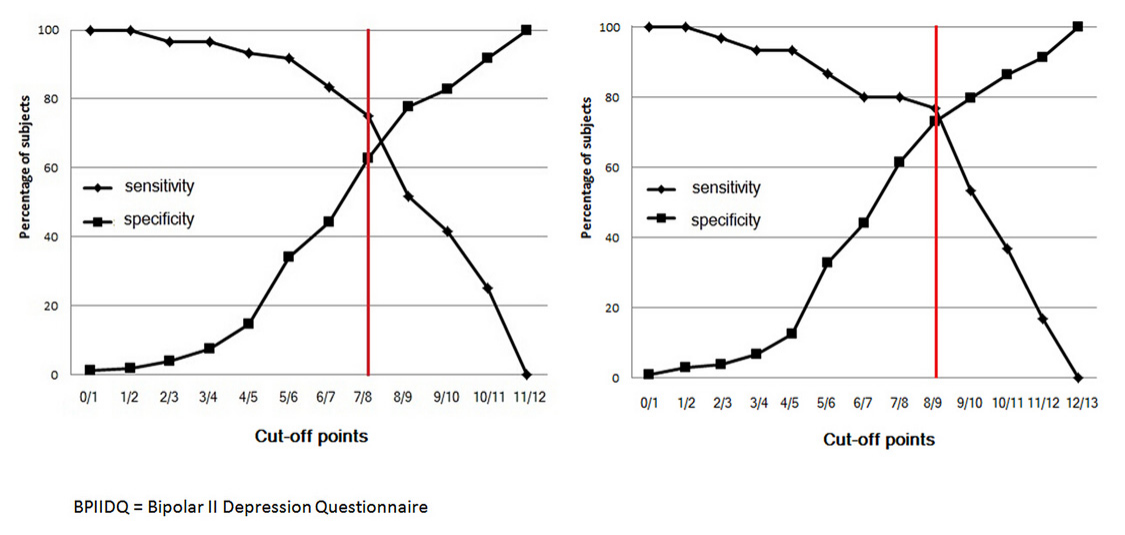
2a. ROC curve for the 8-item BPIIDQ (all cases). Fig 2b. ROC curve for the 8-item BPIIDQ–(females with history of childbirth).

Based on the results of the discriminant analysis and chi-square test results, bdq items 1, 2, 4 and 10, together with bdq items 3 (postpartum depression), 7, 8, 12 and 13 (panic attacks, social phobia, agoraphobia and obsessive rumination, respectively) constituted the final BPIIDQ, with scoring of individual items weighted according to the odds ratios of the respective items. ROC curves were used to explore different combinations of items and weights for optimal sensitivity and specificity (Fig 1A and 1B). The best scoring method was achieved by summing bdq1 x 3, bdq2 x 1, bdq4 x 2, bdq10 x 2, bdq7 x 1, bdq8 x 1 and bdq12 x 1 (7 items), which gave an optimal sensitivity of 0.75 (95%CI 0.62–0.85) and specificity of 0.63 (95%CI 0.55–0.70) (AUC = 0.72), with a cut-off point of 7/8 for the whole sample (Fig 2A). The same scoring method including bdq3 x 1 (8 items) gave an optimal sensitivity of 0.77 (95%CI 0.57–0.89) and specificity of 0.72 (95%CI 0.62–0.80) (AUC = 0.72), with a cut-off point of 8/9 for females with a history of childbirth (Fig 2B). The 8-item BPIIDQ (BPIIDQ-8) and its scoring method are shown in S2 Table.

#### Internal consistency and test-retest reliability

Cronbach’s alpha for the BPIIDQ-8 was 0.60. The mean test-retest reliability coefficient was 0.66 at 2 weeks, with individual values of bdq1 = .85, bdq2 = .55, bdq3 = .82, bdq4 = .39, bdq7 = .76, bdq8 = .46, bdq10 = .90 and bdq12 = .61. The item probing the recurrent nature of BP-II, bdq4, had the lowest score.

#### Item combinations with best specificity

Disregarding sensitivity, the different combinations of bdq items that yielded the best specificity are listed in Table 4. The four bdq items 1, 2, 4 and 10 yielded a specificity of 0.90 in differentiating BP-IID from UPD, while the best specificity obtained with the minimum number of items was 0.92, with bdq1, 2, 4, 10 and 3 (postpartum depression as an addition).

**Table 4:** Bipolar depression question item combinations yielding best specificity.

		Specificity with items scoring positive
No. of items	Best bdq combination	BP-IID against UPD
1	1	0.63
2	1, 2	0.82
3	1, 2, 10	0.87
4	1, 2, 10, 4	0.90
5	1, 2, 10, 4, 3	0.92
6	1, 2, 10, 4, 3, 7	0.92
7	1, 2, 10, 4, 3, 7, 8	0.92

bdq = bipolar depression question

bdq1 = “positive family history”

bdq2 = “onset <25”

bdq3 = “postpartum depression”

bdq4 = “episodic course”

bdq7 = “panic attacks”

bdq8 = “social phobia”

bdq10 = “hypersomnia”

#### Factor analysis

**Fig 3:**
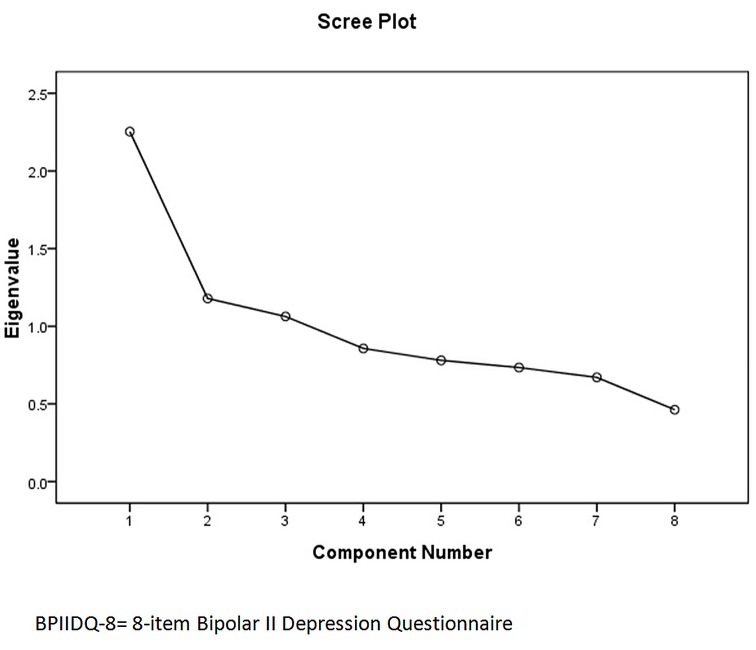
Scree plot for BPIIDQ-8.

Factor analysis of the BPIIDQ-8 with Varimax rotation yielded three factors with Eigenvalues above one, accounting for 56.0% of the variance (Fig 3 and Table 5).

**Table 5:** Rotated component matrix of BPIIDQ-8.

		Component	
	1	2	3
bdq1		.430	
bdq2		.683	
bdq3		.785	
bdq4			.911
bdq7	.546		
bdq8	.806		
bdq10	.659		
Bdq12	.741		

BPIIDQ-8 = 8-item Bipolar II Depression Questionnaire

bdq1 =“positive family history” bdq7 =“panic attacks”

bdq2 =“onset <25” bdq8 =“social phobia”

bdq3 =“postpartum depression” bdq10 = “hypersomnia”

bdq4 =“episodic course” bdq12 =“agoraphobia”

## Discussion

To the best of our knowledge, this study was the first to develop a self-report questionnaire to screen for BP-IID. Investigations that do not distinguish between BP-I and II often yield confusing results. A clinically significant conversion rate from UPD to BP and from BP-II to BP-I [22, 51–53] adds to the confusion. Among the major subtypes, BP-II most easily escapes detection because it cannot be fully confirmed without a definite hypomanic swing that may only emerge over time. The fact that hypomanic episodes in depressed patients are not always correctly identified by clinicians is another reason. In view of the high rate of misdiagnosis and high prevalence of BP-II in the community, this study aimed to construct a simple self-report tool with a reasonable degree of reliability and validity to screen for patients at high risk of developing BP-IID in outpatient settings, including primary care.

Much difficulty was encountered in designing the first four items of the BPIIDQ-P when building the primary test. Item 1 probes for a family history of mood or BP disorder. It was phrased as ‘having a first-degree relative who received psychiatric treatment or died of suicide’. Alternative phrasing, such as a family history of mental illness or mood disorder, was considered too ill-defined, whereas bipolar disorder or mania were considered too specific, particularly in cultures where mental illness is associated with a strong social stigma. Furthermore, misdiagnosis of bipolar disorder as schizophrenia is not uncommon [54]. The chosen phrasing was considered the best compromise with regards to emphasizing familial clustering of mental disorders and suicide in BP-II [41]. Item 2 probes for the age at onset. Most studies put the mean age of onset of BP-II at 20 to 25 [1, 55]. The higher end of the range, 25 years, was chosen to increase the sensitivity and minimize contamination by adolescent turmoil. This cut-off for age at onset is also in line with what has been proposed to differentiate between BP and UPD [27, 56]. Item 3 probes for postpartum depression with the simple statement, “You suffered from postpartum depression”. The severity of the condition is deliberately not qualified, so as to enhance the sensitivity of the question and allow subjects to make their own interpretation. As postpartum mood disorders could be easily missed as postpartum blues [57], respondents’ subjective experience could be a more sensitive guide than a history of medical consultation. Item 4 probes for the episodic nature of BP-II, which most respondents found difficult to recognize, either because they did not have the experience, or because long-lasting low grade fluctuations have become part of their lives. Their uncertainty is reflected in the low test-retest reliability of the response.

Items probing family history, onset before age 25, episodic course and hypersomnia differentiated BP-IID from UPD with p < .05. These differentiating factors are well documented [35, 39] and form the “core” items of the BPIIDQ. Family history of psychiatric treatment or suicide was the only independent predicting or differentiating factor and was identified as the “essential” item. The other items, postpartum depression, panic attacks, social phobia and agoraphobia, that did not distinguish BP-IID from UPD, were designated as ‘accessory’ items. As the constitution of UPD subgroups varies from sample to sample, these “accessory” items were retained in the final version of the questionnaire as guided by the discriminant analysis. They proved to be useful in making the questionnaire more sensitive and specific (Fig 1A and 1B).

BP-IID was similar to recurrent UPD in terms of family history and age at onset, in addition to the recurrent or episodic course. This highlights the unique status of recurrent UPD in relation to BP-II, with its bipolar “flavour” and its difference from other forms of UPD [41, 58]. Although BP-II and BP-I are both classified as BP, the former has been associated with a stronger family history and higher incidence of postpartum depression [21, 59, 60].

In agreement with some [38] but not most [31, 39] findings, no difference was found in the incidence of suicide attempts between BPD and UPD, or between BP-I and BP-II. In addition to sampling and cultural differences across studies, varying definitions of suicide attempts and different ways of collecting information may explain the conflicting results. These findings and considerations justify omitting suicide attempts as a discriminating feature between BP-IID and UPD.

After repeated trials, the BPIIDQ-8, with appropriate weighting factors and adjustment, gave an optimal sensitivity of 77% and specificity of 72% at the cut-off point of 8/9 for females with a history of childbirth. With the postpartum item taken out, the questionnaire yielded a sensitivity of 75% and specificity of 63% at the cut-off point of 7/8 for the whole sample. The psychometric properties of the BPIIDQ-8 are considered satisfactory as a screening tool for specific psychiatric diagnoses [61], thus its external validity is established.

Subgroups such as females without a history of childbirth and males were not treated separately. The former presented with a less satisfactory ROC curve, probably due to their younger age and shorter duration of illness, while the latter comprised a small sample size. With bdq1 (family history) or bdq1 and bdq2 (family history, and onset before 25), the specificity was 66% and 83%, respectively, making it a very simple and easy way to assess the probability of a BP-IID in primary care. If all of the core items, bdqs 1, 2, 4 and 10, score positive, the specificity reaches 89%. The mnemonic ‘FARS’ standing for Family history, Age at onset, Recurrent course and Sleeping too much, could be an easy way to remember.

Apart from satisfactory sensitivity and specificity, BPIIDQ-8 has excellent construct validity, with factor analysis showing three factors with Eigenvalues above one. The first is the “symptom” factor as it contains the classic symptoms of BP-IID including anxiety, hypersomnia and social and agoraphobia. The second is the “biological” factor as it contains items on genetic loading, age at onset and postpartum presentation, thus signifying the possible biological nature of BP-IID. The third factor contains only one item, the “course” factor, which describes the cyclical or episodic nature of BP-II. This agrees well with the theoretical construct as originally conceived.

### Limitations of the study

This study has a number of limitations, the intrinsic one being related to the self-report nature of the BPIIDQ. Further, patients with a history of suicide attempts were excluded because of the low validity of self-report measures [44]. Because suicide attempts are fundamental indicators of BP, strict definitions of suicide attempts should be used in future studies. Recall bias could have been aggravated by current mood disturbances [62, 63], although care was exercised to exclude respondents with significant depressive symptoms. Past experiences such as age at onset and the episodic course of the illness may have been inconsistently reported, as evidenced by their relatively low test-retest reliability.

Another limitation inherent in any study of BP arises from the spectrum nature of BP. UPD is also heterogeneous. The label of UPD is provisional and revised to BP when a history of hypomania is revealed or a hypomanic episode newly emerges. This makes the boundary between BP and UPD indistinct at the clinical level, a fact that undermines the very goal of this study, although great efforts were made to ensure the accuracy of the diagnoses by long-term follow-up and using multiple independent assessors.

The relatively small sample size of only 70 BP-II subjects represents another major drawback, as larger samples would have been needed to clarify the significance of minor differences. This is especially true with the diagnostic value of postpartum depression, as Hong Kong has one of the lowest fertility rates in the world and local women conceive late [64]. A further limitation was that the sample did not include subjects with the diagnostic category of Depression NOS or mixed depression. Mixed depression, defined as depression with symptoms of excitement, is common in both BP and UPD, and is usually associated with antidepressant treatment [65]. For logistical reasons data on the history of and current psychotropic drug treatment were not recorded. The sample was significantly sex-biased, comprising predominantly females. Female predominance in mood disorders is universal but such a high ratio in BP-II is unexpected [66–68]. Under-diagnosis in males and their reluctance to seek help to save face in a paternalistic Chinese society may explain the phenomenon. Finally, the study was conducted at a specialist centre, thus the results may not be generalizable to other settings. The threshold or cut-off points on the scale will need further investigation if used in different cultures.

### Strength of the questionnaire

The inclusion of items was guided by a literature review and adjusted on the basis of statistical analyses. The BPIIDQ-8 is a self-report, single sheet, paper and pencil test that can be completed in 1–2 minutes. Its validation in different types of mood disorder was explored both cross-sectionally and longitudinally against best estimate lifetime diagnoses made by two experienced psychiatrists. Subjects with doubtful diagnoses were independently scrutinized by another psychiatrist using the SCID [9]. The cut-off points were based on optimal sensitivity and specificity assessed by ROC curves [27].

## Conclusions

BP-II is a distinct subgroup of the mood disorder spectrum that poses a diagnostic challenge in both psychiatric and primary care settings. The BPIIQQ-8 is a simple instrument that is able to differentiate BP-IID from UPD with satisfactory sensitivity and specificity. It has good potential as a screening tool for BP-IID in psychiatric outpatient clinics and even in primary care. Although not diagnostic in its own right, a positive result is an indication of the need for more thorough evaluation of BPD.

## Supporting Information

S1 Table Bipolar II Depression Questionnaire—prototype (BPIIDQ-P). (DOC)

S2 Table Bipolar II Depression Questionnaire-8 item (BPIIDQ-8). (DOC)
