# Supplementary material for: The Bipolar II Depression Questionnaire: A Self-Report Tool for Detecting Bipolar II Depression
Source: PLoS One. 2016 Mar 10;11(3):e0149752. doi: 10.1371/journal.pone.0149752 (PMC4786156; doi:10.1371/journal.pone.0149752)
Supplement: S2 Table — (DOC) [file pone.0149752.s002.doc]

S2 Table.

**Bipolar II Depression Questionnaire-8 item**

**(BPIIDQ-8)**

Name: Sex

You have been asked to fill in this questionnaire because you have experienced low mood in the past.

Please answer the following questions by circling “yes” or “no”.

(A) Past History (personal and family history)

1. You have a first degree relative (parent, child, or sibling) who received psychiatric treatment Yes No

or died of suicide.

1. You experienced prominent depressive mood before the age of 25. Yes No
2. You suffered from postpartum depression. Yes No

(Women without history of childbirth please tick this box □)

(B) Past Psychiatric History

1. Your depression is intermittent or episodic in nature, and in between episodes you recover. Yes No
2. In a depressive episode, you feel a nameless unease or fear. Yes No
3. In a depressive episode, you do not want to see anyone. Yes No
4. In a depressive episode, you stay in bed all day and cannot get up in the morning. Yes No
5. In a depressive episode, you dare not leave home or are afraid to go to crowded places. Yes No

The End

Scoring method:

yes = 1 no = 0

Female with history of childbirth:

item 1= x3

2= x1

3= x1

4= x2

5= x1

6= x1

7= x2

8= x1

Total= (statistical cut-off =8/9)

---------------------------------------------------------------------

Others:

item 1= x3

2= x1

3= x0

4= x2

5= x1

6= x1

7= x2

8= x1

Total= (statistical cut-off =7/8)
